# HDACi inhibits liposarcoma via targeting of the MDM2-p53 signaling axis and PTEN, irrespective of p53 mutational status

**DOI:** 10.18632/oncotarget.3230

**Published:** 2015-03-23

**Authors:** Wen-Bin Ou, Jiaqing Zhu, Grant Eilers, Xuhui Li, Ye Kuang, Li Liu, Adrián Mariño-Enríquez, Ziqin Yan, Hailong Li, Fanguo Meng, Haimeng Zhou, Qing Sheng, Jonathan A. Fletcher

**Affiliations:** ^1^ College of Life Sciences, Zhejiang Sci-Tech University, Hangzhou, China; ^2^ Zhejiang Provincial Key Laboratory of Applied Enzymology, Yangtze Delta Region Institute of Tsinghua University, Jiaxing, Zhejiang, China; ^3^ Department of Pathology, Brigham and Women's Hospital and Harvard Medical School, Boston, MA, USA

**Keywords:** liposarcoma, HDACi, MDM2 amplification, PTEN, p53 mutation

## Abstract

The MDM2-p53 pathway plays a prominent role in well-differentiated liposarcoma (LPS) pathogenesis. Here, we explore the importance of MDM2 amplification and p53 mutation in LPS independently, to determine whether HDACi are therapeutically useful in LPS. We demonstrated that simultaneous knockdown of MDM2 and p53 in p53-mutant LPS lines resulted in increased apoptosis, anti-proliferative effects, and cell cycle arrest, as compared to either intervention alone. HDACi treatment resulted in the dephosphorylation and depletion of MDM2 and p53 without affecting CDK4 and JUN expression, irrespective of p53 mutational status in MDM2-amplified LPS. In control mesothelioma cell lines, HDACi treatment resulted in down-regulation of p53 in the p53 mutant cell line JMN1B, but resulted in no changes of MDM2 and p53 in two mesothelioma lines with normal MDM2 and wild-type p53. HDACi treatment substantially decreased LPS and mesothelioma proliferation and survival, and was associated with upregulation of PTEN and p21, and inactivation of AKT. Our findings indicate that wild-type p53 depletion by HDACi is MDM2 amplification-dependent. These findings underscore the importance of targeting both MDM2 and p53 in LPS and other cancers harboring p53 mutations. Moreover, the pro-apoptotic and anti-proliferative effect of HDACi warrants further evaluation as a therapeutic strategy in MDM2-amplified LPS.

## INTRODUCTION

Liposarcoma (LPS) is the most common human sarcoma, representing 24% of extremity and 45% of retroperitoneal soft tissue sarcoma [1–3]. LPS is subdivided into five histopathologic subtypes, including well-differentiated (WDLPS, ~50%), dedifferentiated (DDLPS, 9% to 18%), round cell, myxoid, and pleomorphic. Dedifferentiated, round cell and pleomorphic LPS are high-grade, aggressive tumors with significant metastatic potential while WDLPS and myxoid LPS are low-grade tumors that follow a more indolent clinical course [1, 2, 4]. Although complete surgical resection can be curative, WDLPS often develops in difficult locations such as the retroperitoneum or mediastinum, making complete surgical resection difficult, causing significant complications and high mortality [1, 5]. There are currently no systemic therapeutic regimens known to improve survival in unresectable LPS, underscoring the need for an improved molecular understanding to develop effective targeted therapies.

Regions of chromosome 12q13–15, typically involving *MDM2*, *CDK4*, and *HMGA2*, as well as other genes, are often amplified in WDLPS and DDLPS [6–11]. Additionally, *JUN* maybe amplified in WDLPS cases with a dedifferentiated component [12], and down-regulation or complete loss of *PTEN* or an alternative mechanism of *PIK3CA* mutation results in AKT activation in a subset of LPS [2, 13, 14], implicating the PI3K/AKT/mTOR pathway as a therapeutic target [15]. Dysregulation of the MDM2-p53 regulatory feedback loop is crucial in WDLPS pathogenesis, with most LPS harboring either MDM2 amplification or p53 mutation [6, 7, 16, 17] and individuals with germ-line p53 mutations are at an increased risk of WDLPS development at an earlier age [18]. Inhibition of the MDM2-p53 interaction by Nutlin-3, an antagonist of MDM2, induces apoptosis and growth arrest in p53 wild-type LPS [19, 20].

Histone acetylation is an important determinant of gene expression, and histone deacetylases (HDACs) play a crucial role in cell development and cancer by deacetylating histones and others proteins [21–23]. Numerous studies have demonstrated aberrant expression of HDACs in human cancers, and expression of various HDACs can serve as molecular biomarker of tumors [23, 24]. Overexpression of individual HDACs was able to predict poor patient prognosis independent of tumor type and disease in several cancers [25–28]. Aberrant HDAC activity is linked to key oncogenic events of tumorigenesis [21, 22], and HDAC inhibitors (HDACi) can induce tumor cell apoptosis, senescence, differentiation, cell cycle arrest, and immunogenicity [23]. The HDACi vorinostat and romidepsin have received FDA approval for use against refractory cutaneous T cell lymphoma, and many other HDACi are in clinical trials [23, 29, 30]. The results are promising, but HDACi have pleiotropic effects, acting with various mechanisms across different tumor types, and therefore are not well-understood [21]. However, Blagosklonny and colleagues found that HDACi (FR901228 and trichostatin A) were cytotoxic to cancer cells via depletion of mutant p53 [31], and restored expression and function of pseudo-null p53 [32]. An additional study showed preferential cytotoxicity of an HDACi, SAHA, in mutant p53 cancer cell lines by destabilizing mutant p53 through inhibition of the HDAC6-HSP90 chaperone axis [33]. Functional inactivation of MDM2 and CHIP (carboxy-terminus of HSP70-interacting Protein) by HSP90 results in aberrant stabilization of mutant p53 [34]. Thus, we hypothesized that HDAC inhibition might be therapeutically useful in p53-mutant, Nutlin-3-resistant LPS.

In this study, we evaluate the effects of the HDACi SAHA and LBH589 on proliferation and survival of LPS and control mesothelioma cell lines. We examine the effects of HDACi on amplified MDM2, wild-type and mutant p53, PTEN and AKT. We also demonstrate that dual targeting of amplified MDM2 and mutant p53 shows additive anti-proliferative effects, as compared to either intervention alone. These studies suggest that HDACi warrant clinical evaluation as a therapeutic strategy in LPS harboring mutant p53.

## RESULTS

### Expression of MDM2 and p53, and the anti-proliferative effects of Nutlin-3 in liposarcoma cell lines

**Figure 1:**
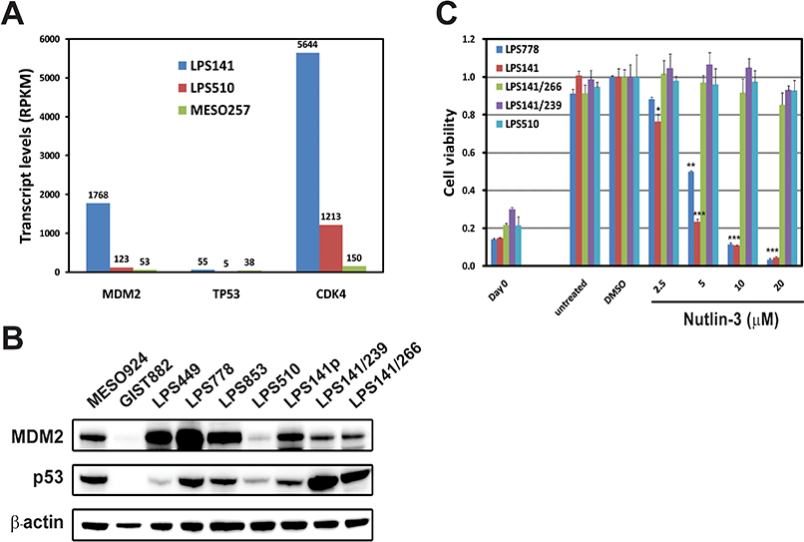
Expression of MDM2 and p53, and the anti-proliferative effects of Nutlin-3 in liposarcoma cell lines **(A)** *MDM2, TP53* and *CDK4* expression in two liposarcoma cell lines (LPS141 and LPS510) and a mesothelioma cell line (MESO257) from whole transcriptome sequencing. RPKM denotes Reads Per Kilobase of exon model per Million mapped reads. **(B)** Immunoblotting demonstrates expression of MDM2 and p53 in liposarcoma cell lines. MESO924 and GIST882 are control lines. **(C)** Viability of liposarcoma cell lines (LPS778, LPS141, LPS141/266, LPS141/239, and LPS510) after 72 h treatment with MDM2 inhibitor Nutlin-3 was evaluated by using the CellTiter-Glo assay. The data were normalized to the DMSO control, and represent the mean values (± s.d.) of quadruplicate cultures. Statistically significant differences between untreated control and treatments are presented as *p < 0.05, **p < 0.01, ***p < 0.001.

Whole transcriptome sequencing at > 25 million mappable reads demonstrated little *MDM2* (53) and *CDK4* (150) transcripts in unamplified MESO257, whereas *MDM2* and *CDK4* were expressed abundantly in LPS141 and LPS510, with 1768 and 123 *MDM2,* and 5644 and 1213 *CDK4* transcript reads, respectively. *TP53* transcript was low in LPS510, which contains a *TP53* point mutation, and normal in *TP53* wild-type LPS141 and MESO257. *MDM2*, *TP53* and *CDK4* transcript levels, expressed as RPKM values, are shown in Figure 1A.

MDM2 protein expression was strong in LPS449, LPS778, LPS853, and LPS141, medium in LPS141/239 and LPS141/266, and weak in LPS510 (Figure 1B). p53 protein expression was strong in p53 mutant LPS141/239 and LPS141/266 and weak in p53 wild-type LPS449 and LPS510. By contrast, expression of MDM2 and p53 was undetectable in GIST882. MDM2 expression in LPS141/239 and LSP141/266, and p53 expression in the liposarcoma lines with wild-type p53 (LPS778, LPS853, and LPS141) were comparable to that in MESO924 cells (Figure 1B).

The effect of Nutlin-3 treatment on cell proliferation is shown in Figure 1C. Nutlin-3 dramatically inhibited cell proliferation in p53 wild-type LPS778 and LPS141 in a concentration-dependent manner. By contrast, Nutlin-3 had only minor effects (5–15% reduction in proliferation) in p53 mutant LPS141/239, LPS141/266, and LPS510, indicating that p53 mutation is a major Nutlin-3 resistant mechanism.

### Biologic effects of HDAC inhibition in LPS and mesothelioma cell lines

#### Degradation of MDM2 and mutant p53, and induction of PTEN by HDACi

**Figure 2:**
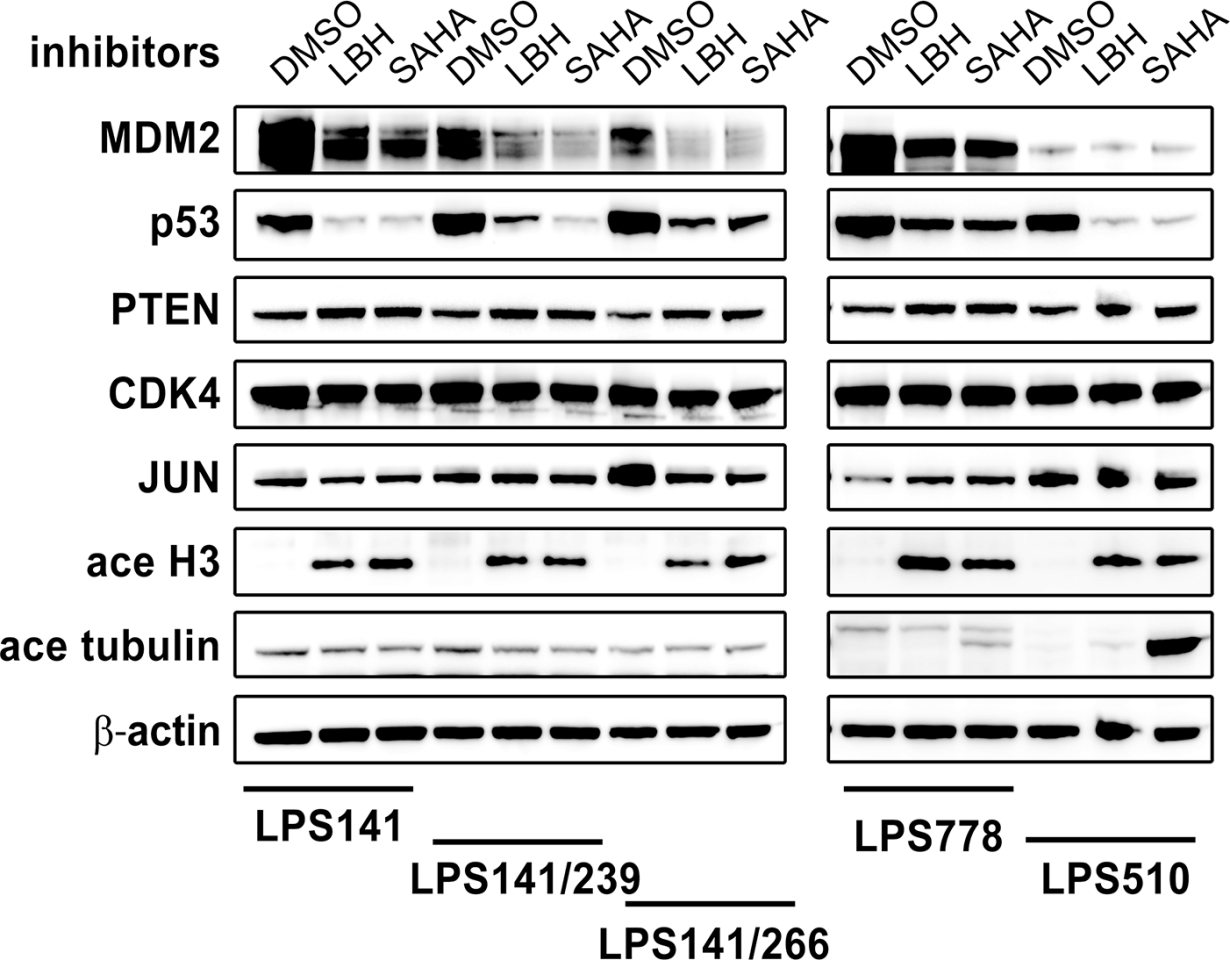
Immunoblotting evaluation of the effect of HDAC inhibitors (100 nM LBH589 and 5 μM SAHA) on expression of MDM2, p53, PTEN, CDK4 and JUN in liposarcoma total cell lysates after 48 hours of treatment in serum-containing medium Acetyl-Histone 3 and Acetyl-tubulin are two acetylation biomarkers. β-actin stain is a loading control.

We evaluated the effects of HDACi on MDM2, p53, PTEN, CDK4, and JUN by immunoblotting in LPS lines by treating with HDACi (LBH589 and SAHA) for 48 hours (Figure 2). LBH589 (100 nM) and SAHA (5 μM) depleted MDM2 and p53, and induced PTEN and acetyl H3 in all LPS lines (Figure 2). Treatment with LBH589 and SAHA had little impact on CDK4 and JUN. HDAC inhibition induced acetyl tubulin in LPS778 and LPS510, but showed little effect in LPS141 and isogenic LPS141/239 and LPS141/266 (Figure 2).

### HDAC regulation of LPS and mesothelioma proliferation and survival

**Figure 3:**
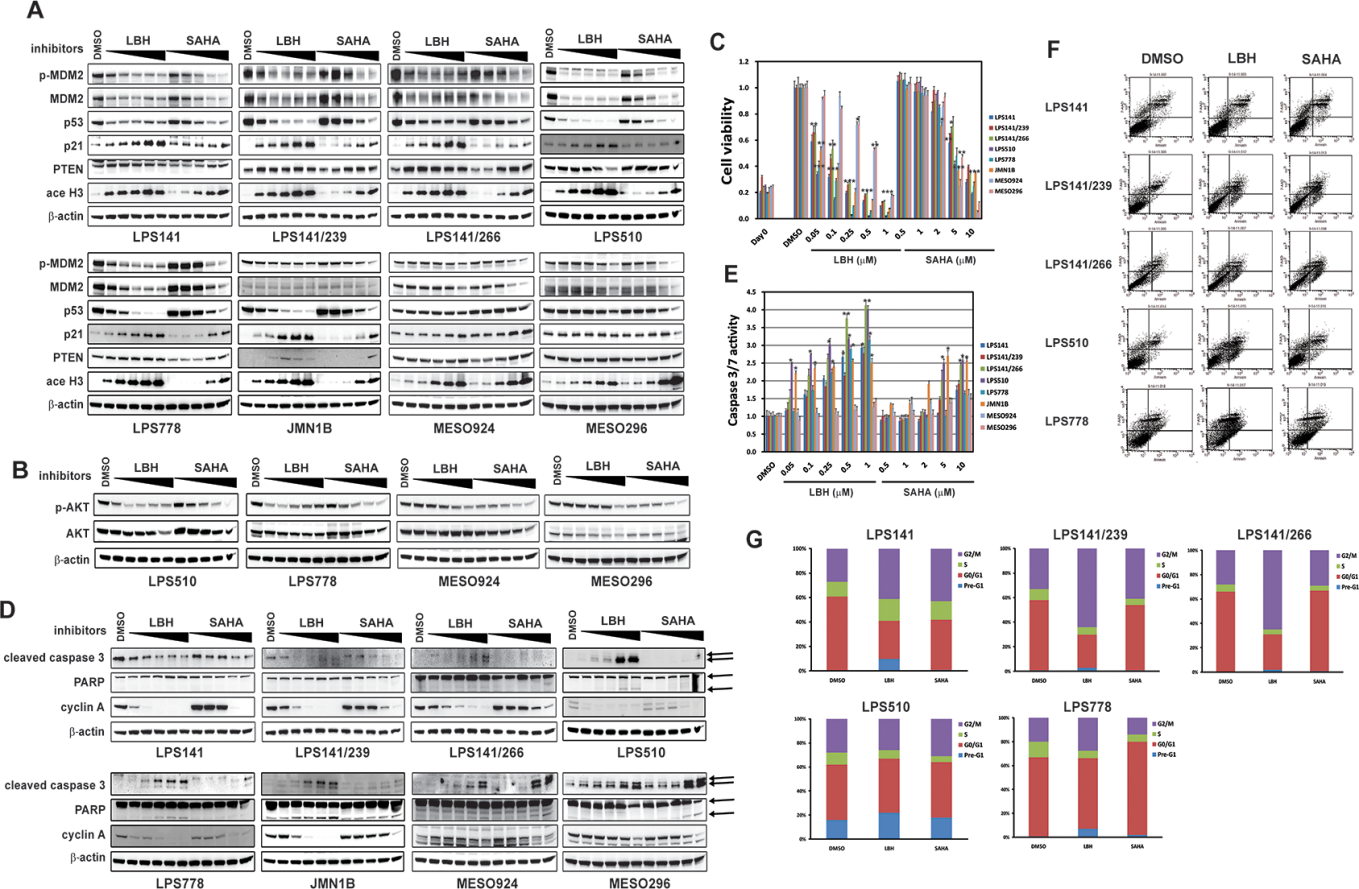
Biologic effects of HDAC inhibition in liposarcoma and mesothelioma cell lines **(A)** Effects of LBH589 (50, 100, 250, 500 and 1000 nM) and SAHA (0.5, 1, 2, 5, 10 μM) on expression of phospho-MDM2, MDM2, p53, p21, and PTEN in liposarcoma (LPS141, LPS141/239, LPS141/266, LPS510, and LPS778) and mesothelioma (JMN1B, MESO924, and MESO296) total cell lysates, after 48 hours of treatment. Acetyl-Histone 3 is an acetylation biomarker. β-actin stain is a loading control. **(B)** Immunoblotting assays evaluating the effects of LBH589 (50, 100, 250, 500 and 1000 nM) and SAHA (0.5, 1, 2, 5, 10 μM) on expression of phospho-AKT and AKT in LPS510, LPS778, MESO924, and MESO296, after 48 hours of treatment. β-actin stain is a loading control. **(C)** Liposarcoma cell (LPS141, LPS141/239, LPS141/266, LPS510, and LPS778) and mesothelioma cell (JMN1B, MESO924, and MESO296) viability after 72 hours of treatment with inhibitors including LBH589 (50, 100, 250, 500 and 1000 nM), and SAHA (0.5, 1, 2, 5, 10 μM), using the CellTiter-Glo assay. The data are normalized to the DMSO control, and represent the mean values (± s.d.) of quadruplicate cultures. Statistically significant differences between untreated control and treatments are presented as *p < 0.05, **p < 0.01, ***p < 0.001. **(D)** Immunoblotting assays evaluating the effects of HDCAi on apoptotic markers (cleaved caspase 3 and PARP), and proliferation markers (Cyclin A). Actin stain serves as a loading control. **(E)** Apoptosis after HDACi treatment for 48 hours in serum-containing medium. Caspase 3/7 activity was measured using a Caspase-Glo luminescence assay; data were normalized to the DMSO control, and are shown as mean values (± s.d.) from quadruplicate cultures. Statistically significant differences between untreated control and treatments arepresented as *p < 0.05, **p < 0.01, ***p < 0.001. **(F)** Apoptosis analyses following LBH (250 nM) and SAHA (5 μM) treatment for 48 hours using PE Annexin V Apoptosis Detection Kit I. **(G)** Cell cycle analyses after 48 hours of inhibitor (250 nM LBH589 and 5 μM SAHA) treatment in serum-containing medium.

HDACi LBH589 and SAHA were evaluated by immunoblotting, apoptosis, proliferation, and cell cycle analyses (Figure 3A–3G). Treatment with HDACi LBH589 and SAHA induced expression of p21, PTEN, and acetyl-H3 in all LPS lines (Figure 3A), and inactivated AKT in LPS510, LPS778, MESO924, and MESO296 (Figure 3B) in a concentration-dependent manner. Treatment with HDACi resulted in degradation of mutant p53, and induction of p21, PTEN, and acetyl-H3, but had little effect on phosphorylation and expression of MDM2 in JMN1B. HDACi LBH589 and SAHA showed minimal effect on expression/phosphorylation of MDM2 and p53 in MESO924 and MESO296 (Figure 3A).

Cell proliferation was strongly inhibited in all LPS and mesothelioma cell lines in a dose-dependent manner after HDAC inhibition (Figure 3C): LBH589 and SAHA IC_50_ values in p53-mutant lines were 66 nM and 6.5 μM (LPS141/239), 110 nM and 7.1 μM (LPS141/266), 34 nM and 4.2 μM (LPS510), and 52 nM and 3.7 μM (JMN1B), respectively, and in wild-type p53 lines were 59 nM and 5.8 μM (LPS141), 41 nM and 4.2 μM (LPS778), 520 nM and 3.8 μM (MESO924), and 540 nM and 4.7 μM (MESO296), respectively. The anti-proliferative effect of LBH589 was stronger in all LPS cell lines and p53 mutant JMN1B than in p53 wild-type MESO924 and MESO296, whereas the anti-proliferative effect was greater in MESO924 and MESO296 than JMN1B and all LPS cell lines after treatment with SAHA (Figure 3C). HDAC inhibition also dramatically suppressed the expression of cyclin A in all five LPS lines and JMN1B, but moderately inhibited cyclin A expression in MESO924 and MESO296 (Figure 3D).

Treatment with LBH and SAHA for 48 hours induced apoptosis in all cell lines, as evidenced by increased expression of caspase 3, increased caspase 3/7 activity (Figure 3D and 3E), and PARP cleavage (Figure 3D), in a dose-dependent manner. The apoptotic effects of LBH589 were stronger in all LPS and JMN1B than those of SAHA. Caspase 3/7 activity was more dramatic in LPS and mesothelioma cell lines with MDM2 amplification and/or p53 mutation than in cell lines with wild-type p53 (Figure 3E). LPS cell lines analyzed 48 hours after LBH589 and SAHA treatments showed a dramatic increase in apoptotic cells compared to vehicle-treated cells, particularly in lines harboring mutant p53 (Figure 3F and Supplementary Table 1). Apoptosis was most prominent in LPS141 and LPS510, with nuclear fragmentation increasing from 0% (LPS141), 16% (LPS510), and 1% (LPS778) in cells treated with DMSO, to 10% (LPS141), 22% (LPS510), and 7% (LPS778) in cells treated with 250 nM LBH589 (Figure 3F and Supplementary Table 1).

Cell-cycle analysis in LPS141, LPS141/266, LPS141/239, and LPS778 showed a G2 block after HDAC inhibition with an increase in the G2 peak from 27%, 28%, 33% and 20% in DMSO control cells to 41%, 65%, 64%, and 27% after LBH589 treatment, respectively (Figure 3G and Supplementary Table 2). Treatment with 5 μM SAHA resulted in G1 arrest in LPS778 (DMSO: 66%; SAHA: 78%), and G2 arrest in LPS141 (DMSO: 27%; SAHA: 43%), LPS141/239 (DMSO: 33%; SAHA: 41%), and LPS510 (DMSO: 28%; SAHA: 31%) (Figure 3G and Supplementary Table 2).

### Additive effect of simultaneous knockdown of MDM2 and mutant p53 in LPS

**Figure 4:**
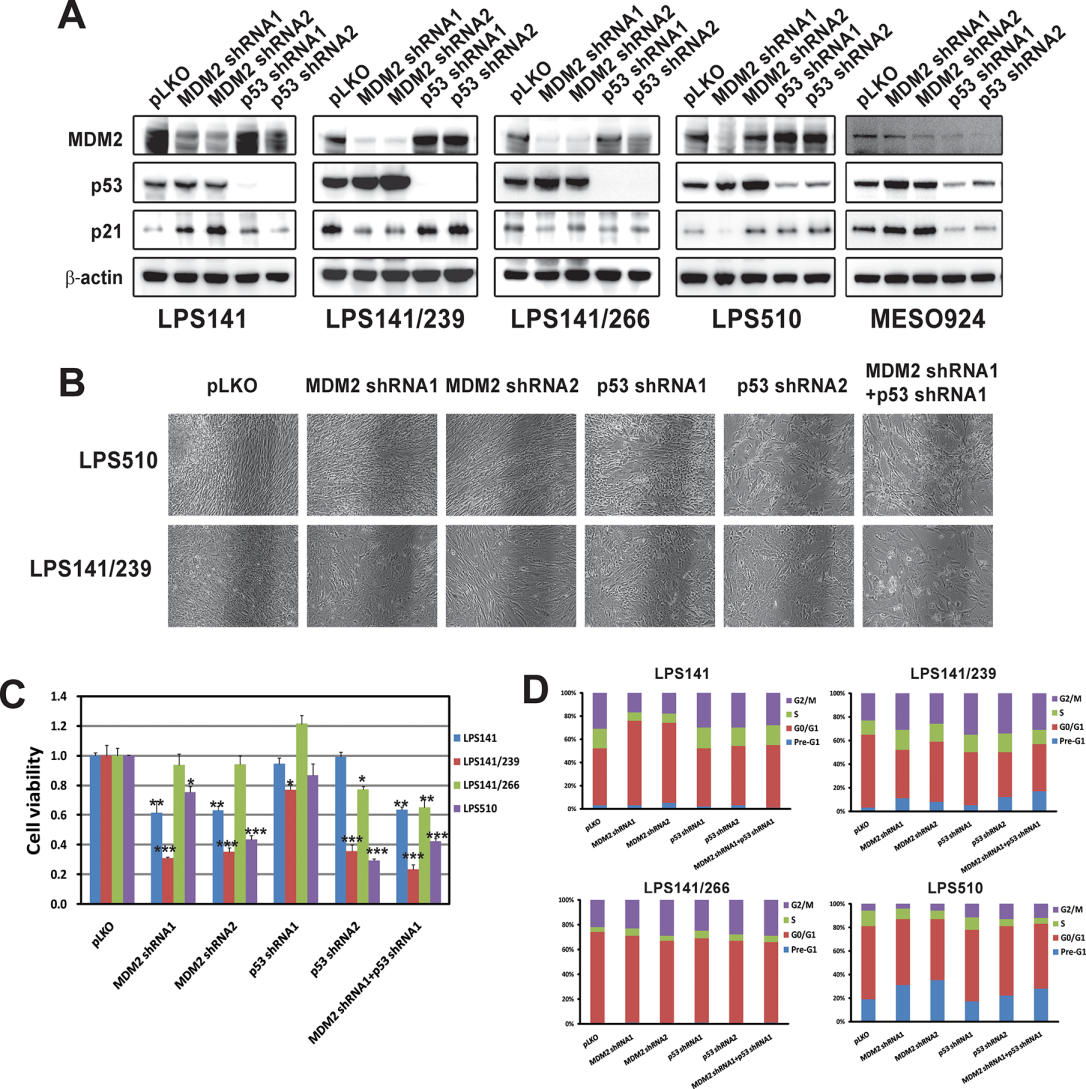
Additive effects were observed through coordinated knockdowns of *MDM2* and *TP53* as demonstrated by immunoblotting (A) cell culture appearance (B) cell viability (C) and cell cycle analyses (D) showing that *MDM2* and *TP53* knockdown had greater anti-proliferative effects and cell cycle arrest, compared to either intervention alone in MDM2-amplified and p53 mutant liposarcoma cell lines (A) MDM2, p53, and p21 were evaluated by immunoblotting at 10 days post-infection with stable *MDM2* and/or *TP53* shRNA expression. Actin staining is a loading control. (B) Cell culture appearance in LPS141/239 and LPS510 at 10 days after infection by lentiviral *MDM2* and/or *TP53* shRNA constructs, showing greater growth inhibition compared to either intervention alone. (C) Cell viability evaluated by a Cell-titer Glo^®^ ATP-based luminescence assay in liposarcoma cell lines (LPS141, LPS141/239, LPS141/266, and LPS510), at 72 hours following stable *MDM2* and/or *TP53* shRNA expression for 10 days. Data were normalized to the empty lentivirus infections or DMSO, and represent the mean values (± s.d.) from quadruplicate cultures. Statistically significant differences between empty vector control and target gene shRNAs are presented as *p < 0.05, **p < 0.01, ***p < 0.001. (D) Cell cycle analyses performed at 72 hours following stable lentiviral *MDM2* and/or *TP53* shRNA expression for 10 days.

*MDM2* and *TP53* gene expression was stably silenced by lentivirus-mediated shRNAs (Figure 4), and knockdown was evaluated by immunoblotting, in LPS141, LPS141/239, LPS141/266, and LPS510, and MESO924, at 10 days after transduction (Figure 4A). MESO924 has normal MDM2 and wild-type p53. We achieved greater than 60% knockdown of the targets with at least one construct. MDM2 knockdown increased p53 and p21 expression, irrespective of p53 mutational status, in p53 wild-type LPS141 and MESO924. p53 knockdown had little effect on MDM2 and p21 in LPS cell lines but decreased MDM2 and p21 expression in MESO924 (Figure 4A). MDM2 or p53 knockdown alone dramatically reduced LPS510 and LPS141/239 cell growth. The combination of MDM2 or p53 knockdown had an additive effect on cell growth, compared to either intervention alone (Figure 4B). MDM2 knockdown, in p53 wild-type LPS141, and p53-mutant LPS141/239 and LPS510, resulted in ~40% and ~60–70% inhibition of cell viability, respectively, at 10 days after MDM2 silencing, compared to the empty vector control. MDM2 knockdown resulted in a mild reduction in viability for a p53 mutant LPS141/266 (Figure 4C). p53 knockdown with at least one construct resulted in 20–70% reduction in viability of three mutant p53 LPS cell lines (LPS141/266, LPS141/239, and LPS510), but had little effect on viability of p53 wild-type LPS141. Combination of MDM2 and p53 knockdown resulted in 40–80% reduction in cell viability and showed a greater effect on cell growth, in three mutant p53 LPS cell lines (LPS141/266, LPS141/239, and LPS510), compared to either intervention alone (Figure 4B).

MDM2 knockdown resulted in a dramatic G1 block in p53-wild-type LPS141, or G2 block in p53-mutant LPS141/266 and LPS141/239. The G1 peak was 49% (LPS141), and the G2 peaks were 22% (LPS141/266), 23% (LPS141/239), and 6% (LPS510) in the empty vector-treated cells compared to 69–73% (LPS141) in G1 populations, and 23–29% (LPS141/266), and 26–31% (LPS141/239) in G2 populations in *MDM2 shRNA1/2*-treated cells (Figure 4D and Supplementary Table 3). Cell cycle analyses also demonstrated a G2 block after p53 knockdown in LPS141/266, LPS141/239, and LPS510 (Figure 4D). The G2 peaks were 25–28% (LPS141/266), 34–35% (LPS141/239), and 12–13% (LPS510) in cells with p53 knockdown (Figure 4D and Supplementary Table 3). Combination of MDM2 and p53 knockdown resulted in a G2 block for LPS141/239, LPS141/266, and LPS510 (Figure 4D). *MDM2*, *p53*, or both *shRNA* knockdown also induced apoptosis in two mutant p53 liposarcoma cell lines (LPS141/239 and LPS510): in LPS141/239, nuclear fragmentation was demonstrated in 3% cells treated with empty vector control, but in 11% cells treated with *MDM2 shRNA1,* in 5% cells treated with *p53 shRNA1*, and in 17% cells treated with combination of *MDM2 shRNA1* and *p53 shRNA1*; in LPS510, nuclear fragmentation was demonstrated in 19% cells treated with empty vector control, but in 31% cells treated with *MDM2 shRNA1,* in 18% cells treated with *p53 shRNA1*, and in 28% cells treated with combination of *MDM2 shRNA1* and *p53 shRNA1* (Figure 4D and Supplementary Table 3).

### Mutant p53 interacts with MDM2

The interaction between MDM2 and p53 was evaluated in LPS cell lines by p53 immunoprecipitation followed by MDM2 immunoblotting. A MDM2 80 kDa band was observed in LPS cell lines irrespective of p53 mutation status (Supplementary Figure 1).

## DISCUSSION

Almost all cases of WDLPS harbor *MDM2* amplification or *TP53* mutations [7, 9, 17]. Amplification of *MDM2* and *CDK4* has been found in WD and DDLPS [6, 7, 35]. Our findings confirm amplification of *MDM2* and *CDK4* in LPS cell lines, as compared to unamplified mesothelioma cell lines (MESO257 or MESO924) (Figure 1A and 1B, and SNP data (not shown)). The crucial oncogenic role of MDM2-p53 has recently been demonstrated by the inhibitory effect of Nutlin-3, an antagonist of MDM2, on *MDM2*-amplified LPS cell lines [19, 20]. In the present work, we confirm that Nutlin-3 inhibits cell viability in two LPS lines harboring wild-type p53 (LPS778 and LPS141), but show an attenuated effect in LPS lines harboring mutant p53 (LPS141/239, LPS141/266, and LPS510) (Figure 1C). This result indicates that p53 mutation is a Nutlin-3 resistance mechanism in LPS.

Control of p53 expression by MDM2 is lost in tumors harboring mutant p53, resulting in p53 hyperstabilization and accumulation [34]. We hypothesized that HDAC inhibition might induce pro-apoptotic and anti-proliferative effects in liposarcoma cell lines harboring p53 mutations. Recent studies have demonstrated functional inactivation of MDM2 and CHIP E3 ligase activity by HSP90 inhibition, resulting in aberrant stabilization of mutant p53 [34], and SAHA inhibited cell growth in mutant p53 human cancer cells by destabilizing mutant p53 through inhibition of the HDAC6-HSP90 chaperone axis [33]. HDACi showed a strong impact on cell viability and apoptosis in LPS and mesothelioma cell lines containing mutant or wild-type p53 (Figure 3C–3G). Treatment with LBH589, in *MDM2*-amplified and/or p53-mutant cell lines (LPS141, LPS141/239, LPS141/266, LPS510, LPS778, and JMN1B), had greater inhibitory effects on viability than in normal MDM2 and p53-wild-type cell lines MESO924 and MESO296, whereas the anti-proliferative effects of HDAC inhibition by SAHA in p53-wild-type mesothelioma cell lines were greater (MESO924 and MESO296) than LPS cell lines (Figure 3C). These data suggest that HDAC regulation of LPS cell viability can be targeted effectively in MDM2-amplified and/or p53-mutant cancer cells by LBH589, and that SAHA treatment can show dramatic anti-proliferative effects in-p53 wild type cancer cells.

We next investigated the effects of HDACi LBH589 and SAHA on the expression of MDM2 and p53 in these cell lines (Figures 2 and 3A). Immunoblotting showed that treatment with HDACi LBH589 and SAHA for 48 hours induced the degradation of mutant p53 in three LPS cell lines (LPS141/239, LPS141/266, and LPS510) and a mesothelioma cell line (JMN1B) (Figures 2 and 3A). This finding is in line with published data showing that SAHA treatment led to degradation of mutant p53 [33]. Unexpectedly, HDAC inhibition by SAHA and LBH589 resulted in down-regulation of wild-type p53 in two LPS cell lines (LPS141 and LPS778), and a decrease in MDM2 and phospho-MDM2 in all LPS cell lines, irrespective of p53 status (Figures 2 and 3A). By contrast, expression and phosphorylation of MDM2, in three normal MDM2 mesothelioma cell lines (JMN1B, MESO924, and MESO296), and expression of p53, in two p53 wild-type mesothelioma cell lines (MESO924 and MESO296), were unchanged or slightly affected after treatment with HDACi (Figures 2 and 3A). Unexpectedly, HDAC inhibition resulted in p53 (wild-type and mutant) degradation in a dose-dependent manner in LPS cell lines, whereas treatment with HDACi induced p21 expression, a direct p53 downstream responder (Figure 3A), indicating that regulation of p21 is p53-independent after HDAC inhibition. These novel findings indicate that HDACi regulation of wild-type p53 and MDM2 is MDM2 amplification-dependent.

*CDK4* and *JUN* are amplified in LPS [12]. Recent studies have demonstrated that the selective cyclin-dependent kinase 4/6 (CDK4)/CDK6 inhibitor PD0332991 inhibits growth and induces senescence in LPS cell lines and xenografts. Furthermore, treatment with the CDK4 inhibitor PD0332991 was associated with a favorable progression-free survival in patients with CDK4-amplified and RB-expressing WDLPS/DDLPS who had progressive disease despite systemic therapy [36]. In addition, JUN knockdown by shRNAs reduced cell viability *in vitro* and inhibited tumor formation *in vivo* without an observable effect on the differentiation state of the LPS cells [12]. Interestingly, in the present report, unlike the effects of HDACi on MDM2 expression (Figure 2), neither LBH589 nor SAHA affected expression of CDK4 or JUN, indicating that the anti-proliferative and pro-apoptotic effects of HDACi do not depend on CDK4 or JUN in LPS cell lines (Figure 2). Therefore, these data indicate that MDM2 amplification and/or p53 mutation play the crucial oncogenic role in LPS and mesothelioma cell proliferation and survival.

Previous studies have suggested that aberrant activation of PI3-K/AKT/mTOR signaling due to down-regulation or complete loss of *PTEN* or an alternative mechanism of *PIK3CA* mutation, is a potential therapeutic target in LPS and mesothelioma [2, 13, 14, 37, 38]. Although the mechanism is unclear, we show here that HDACi LBH589 and SAHA induce PTEN expression in all LPS and mesothelioma cell lines tested (Figure 2 and Figure 3A) accompanied by AKT inhibition (Figure 3B), consistent with published data that NBM-HD-3, a potent HDAC inhibitor, increases PTEN expression [39]. These data suggest that the apoptotic and anti-proliferative effects of HDACi are also associated with up-regulation of PTEN and inactivation of AKT.

We previously reported that MDM2 inhibition (Nutlin-3) induced p53 expression, cell apoptosis, anti-proliferative effects and cell cycle arrest in mesothelioma [40]. In the present work, treatment with HDACi LBH589 and SAHA induced apoptosis and reduced cell viability in two p53 wild-type mesothelioma cell lines (MESO924 and MESO296) and one p53 mutant mesothelioma cell line (JMN1B), associated with an up-regulation of PTEN and p21, inactivation of AKT, and degradation of mutant p53 (Figure 3A–3F). More recent studies also show that SAHA-induced apoptosis is FLIP/caspase 8-dependent and HR23B-independent in mesothelioma [41]. Our data add to the already substantial evidence that targeting the crucial HDACi pathway might be an effective strategy in mesothelioma, especially in p53-mutant sublines.

HDAC inhibition resulted in downregulation of amplified MDM2 and mutant p53, and significantly reduced LPS cell viability (Figures 2, 3A–3G). On the basis of these findings, we hypothesized that additive effects would be obtained through attacking both amplified MDM2 and mutant p53 together. Therefore, we further evaluated the function of the amplified MDM2, mutant p53 alone, or both together, by *shRNA* knockdown. *MDM2* or *p53 shRNA* knockdown in the MDM2-amplified and/or p53-mutant LPS cell lines had profound anti-proliferative consequences (Figure 4B–4D). By contrast, p53 silencing did not affect proliferation in wild-type p53 LPS cell line LPS141 (Figure 4C and 4D). These findings suggest that MDM2 amplification or p53 mutation play essential oncogenic roles in LPS. Notably, additive effects were obtained through simultaneous MDM2 and p53 knockdowns, with this combination approach inhibiting cell growth more than either intervention alone (Figure 4B and 4C). In addition, p53 mutations, including N239D (LPS141/239), H179R (LPS510) or G266R (LPS141/266) did not affect the interaction of MDM2 and p53 (Supplementary Figure 1), and MDM2 knockdown resulted in accumulation of mutant p53, indicating that MDM2 remains a major regulator of mutant p53. Based on the evidence presented in this report, we propose MDM2 amplification and p53 mutation as essential proliferation mediators in LPS, and suggest that targeting HDAC or MDM2 amplification and mutant p53 inhibition is a therapeutic strategy in this challenging subset of LPS.

## MATERIALS AND METHODS

### Antibodies and reagents

Monoclonal antibodies to p53, cyclin A, and polyclonal antibody to CDK4 were from Santa Cruz Biotechnology (Santa Cruz, CA). Monoclonal antibody to acetyl-tubulin, and polyclonal antibodies to phospho-MDM2 (Ser166), PTEN, phospho-AKT (Ser473), AKT, JUN, acetyl-histone H3, and cleaved caspase 3 were from Cell Signaling Technology (Beverly, MA). Antibodies to PARP, MDM2, and p21 were from Invitrogen Laboratories (Invitrogen life Technologies, Carlsbad, CA). Polybrene, puromycin, and antibody to β-actin were from Sigma-Aldrich (St, Louis, MO). Lentiviral shRNA constructs were from The RNAi Consortium (TRC, Cambridge, MA, USA), and included MDM2: CTTTGGTAGTGGAATAGTGAA (shRNA1); CTCAGCCATCAACTTCTAGTA (shRNA2), and p53: CTTCGACTATCTCAAACTCCT (shRNA1), CAAGGTACTTCGATGATGAAT (shRNA2). LBH589 (Panobinostat) and SAHA (vorinostat) were obtained from LC Labs (Woburn, MA). These inhibitors were reconstituted in DMSO.

### Liposarcoma and mesothelioma cell lines

LPS141, LPS141/266, LPS141/239, and LPS510 were developed in the Department of Pathology at Brigham and Women's Hospital. The LPS141 cell line was developed from a DDLPS with heterologous osteosarcoma, arising in a patient with a history of recurrent WDLPS [12]. LPS141/266 and LPS141/239 are isogenic sublines of LPS141 which retain the parental *MDM2* and *CDK4* genomic amplification but have p53 point mutations (G266R and N239D, respectively). LPS510 contains *MDM2* and *CDK4* genomic amplification, and a p53 point mutation (H179R). T778 (LPS778) and LPS449 were a gift from Dr. Florence Pedeutour, were established from a patient with recurrent WDLPS, and are p53 wild-type [42]. Mesothelioma cell lines established in Dr. Jonathan Fletcher's laboratory from epithelial-type mesotheliomas (MESO924 and JMN1B) [43, 44] or mixed histology mesothelioma (MESO296) [44, 45]. MESO924 and MESO296 have wild-type p53, but JMN1B contains a p53 point mutation (G245S). All cell lines were validated by unique clonal cytogenetic aberrations within 10 passages of the present studies (MESO924 and MESO296 were validated by comparison with the corresponding surgical specimens, and JMN1B was validated by comparison with published cytogenetic aberrations).

### Whole transcriptome sequencing

rRNA was depleted from 5 μg of total RNA using biotinylated oligonucleotides (Ribominus, Invitrogen), and libraries were constructed from the rRNA-depleted RNA according to the SOLiD Total RNA-seq Kit Protocol (Applied Biosystems). Briefly, RNAs were fragmented by RNAse III to an average size of 150 bases, ligated to adaptors in a directed orientation, and the resultant library served as template for cDNA synthesis and PCR amplification. Approximately 50 bases were sequenced from one end of each fragment using either the SOLiD 3+ or SOLiD 4 instrument and reagents (Applied Biosystems). The resulting sequence data were mapped to the human reference genome, hg18, using Bioscope v1.2 (Applied Biosystems). Sequences that mapped to unique locations were quantified per transcriptional unit, as defined in RefSeq (release 35), as “Reads Per Kilobase of transcript per Megabase of total sequence (RPKM)”. In addition, a weighted score was used to describe significant disparities between samples for each transcript in a manner incorporating the magnitude of expression as well as the difference.

### Protein lysate preparations and immunoblotting

Immunoblotting was performed after 48 hours of treatment with LBH589 or SAHA, and after 10 days post-infection with *MDM2/p53 shRNAs*. Whole cell lysates were prepared using lysis buffer (1% NP-40, 50 mM Tris-HCl pH 8.0, 100 mM sodium fluoride, 30 mM sodium pyrophosphate, 2 mM sodium molybdate, 5 mM EDTA, and 2 mM sodium orthovanadate) containing protease inhibitors (10 μg/mL aprotinin, 10 μg/mL leupeptin, and 1 mM phenylmethylsulfonyl fluoride). Lysate protein concentrations were determined using a Bio-Rad protein assay (Bio-Rad Laboratories Hercules, CA, USA). Electrophoresis and western blotting were performed as described previously [46]. The hybridization signals were detected by chemiluminescence (Immobilon™ Western, Millipore Corporation, MA) and captured using a GE FUJI ImageQuant LAS4000 chemiluminescence imaging system.

### Immunoprecipitation

Sepharose-protein G beads linked to goat polyclonal antibody were used. One mg of protein lysate (500 μL) was preadsorbed for 30 min using 20 μl of protein G beads at 4°C. 10 μl of primary antibodies against mouse p53 (0.2 μg/μL) were rocked with the lysates for 2 hours at 4°C. Then, 20 μL of sepharose-protein G beads was added and rocked overnight at 4°C, then centrifuged at 10,000 rpm for 2 min at 4°C, after which the sepharose beads were washed 3 times with 750 μL of IP buffer (25 min/each time) and once with 750 μL 10 mM Tris-Cl buffer (pH7.6). Loading buffer (20 μL) was added to the beads and boiled for 5 min at 95°C.

### Preparation of shRNA lentiviruses

Lentiviruses were produced by cotransfecting pLKO.1puro plasmids containing *MDM2* or *p53 shRNAs*, and helper virus packaging plasmids pCMVßR8.91 and pMD.G (at a 10:10:1 ratio) into 293T cells. Transfections were carried out using lipofectamine and PLUS reagent (Invitrogen life Technologies, Carlsbad, CA). Lentiviruses were harvested at 24, 36, 48, and 60 hours post-transfection. Virus was frozen at −80°C in appropriately sized aliquots for infection. Well-validated shRNAs were used for MDM2 and p53 knockdowns.

### Cell culture and virus infection

LPS cell lines (LPS141, LPS141/239, LPS141/266, LPS449, LPS778, and LPS510) and mesothelioma cell lines (MESO924, MESO296, and JMN1B) were maintained in RPMI 1640 with 10% fetal bovine serum (FBS) supplemented with penicillin/streptomycin and 1% (v/v) L-glutamine. LPS cells and MESO924 were seeded in six-well plates and lentiviral shRNA infections were carried out in the presence of 8 μg/mL polybrene. All lentiviral experimental results were performed in duplicate. Following transduction, LPS141, LPS141/239, LPS141/266, LPS510, and MESO924 cells were selected for stable expression of the MDM2 or p53 shRNAs using 2 μg/ml puromycin. Cell culture images were obtained using SPOT software and a Nikon Eclipse TE2000–5 inverted microscope and cells were lysed for western blotting or cell cycle analysis 10 days post-infection.

### Cell proliferation and apoptosis assays

Cell lines were plated at 3,000 cells/well in a 96-well flat-bottomed plate (Falcon, Lincoln NJ) and cultured for 24 hours before treatment with different inhibitors, which included Nutlin-3 (2.5, 5, 10 and 20 μM), LBH589 (0.05, 0.1, 0.25, 0.5 and 1 μM), SAHA (0.5, 1, 2, 5 and 10 μM), *MDM2 shRNAs* or *p53 shRNAs*. Cell viability was determined after treatment with inhibitors or *shRNAs* for 3 or 8 days, respectively, using the CellTiter-Glo luminescent assay (Promega, Madison, Wisc.) and measured using a Veritas™ Microplate Luminometer (Turner Biosystems, Sunnyvale, CA). The data were normalized to the control group (empty vector or DMSO). The IC_50_ value was defined as the concentration that causes 50% growth inhibition. IC_50_ values were calculated using a sigmoidal curve fit with GraphPad Prism Software (GraphPad Software, Inc., La Jolla, CA, USA). All experimental points were set up in four replicate wells and independently performed in duplicate.

Apoptosis was evaluated using the PE Annexin V Apoptosis Detection Kit I (BD Pharmingen™, USA). Briefly, LPS141, LPS141/239, LPS141/266, LPS510, and LPS778 cells in 6-well plates were treated with LBH589 (250 nM) or SAHA (5 μM) for 48 hours, trypsinized and washed twice with cold Hanks Balanced Salt Solution and treated with 5 μl of PE Annexin V and 5 μl 7-AAD in 1X Binding Buffer for 15 minutes at RT (25°C) in the dark. The stained cells were analyzed in a flow cytometer (FACScan, BD Biosciences) within 1 hour and CellQuest software (BD Biosciences) was used to analyze the data.

### Cell cycle analysis

LPS141, LPS141/239, LPS141/266, LPS510, and LPS778 cells in 6-well plates were treated with inhibitors for 48 hours or *MDM2* and *p53 shRNAs* for 10 days with selection, then trypsinized and washed once with ice-cold PBS. For nuclear staining, cells were fixed by 70% ethanol for 24 hours. A PBS solution containing RNase (10 μg/ml) and propidium iodide (PI) (Roche) was added to the cells and incubated for 15 minutes at 37°C. The cell suspension was analyzed on a flow cytometer (FACScan, BD Biosciences) within 48 hours and ModFit LT (Macintosh) was used to analyze the data.

### Statistical analysis

Student's *t*-tests were performed on data from cells treated with control DMSO or inhibitors, as well as cells treated with empty vector, *MDM2*, or *p53 shRNAs*. Statistically significant differences between untreated control and treatment were defined as *P < 0.05, **P < 0.01 and ***P < 0.001.

## SUPPLEMENTARY FIGURE AND TABLES


