# Supplementary material for: HDACi inhibits liposarcoma via targeting of the MDM2-p53 signaling axis and PTEN, irrespective of p53 mutational status
Source: Oncotarget. 2015 Mar 23;6(12):10510–20. doi: 10.18632/oncotarget.3230 (PMC4496371; doi:10.18632/oncotarget.3230)
Supplement: Supplementary file 1 [file oncotarget-06-10510-s001.pdf]

SUPPLEMENTARY FIGURE AND TABLES

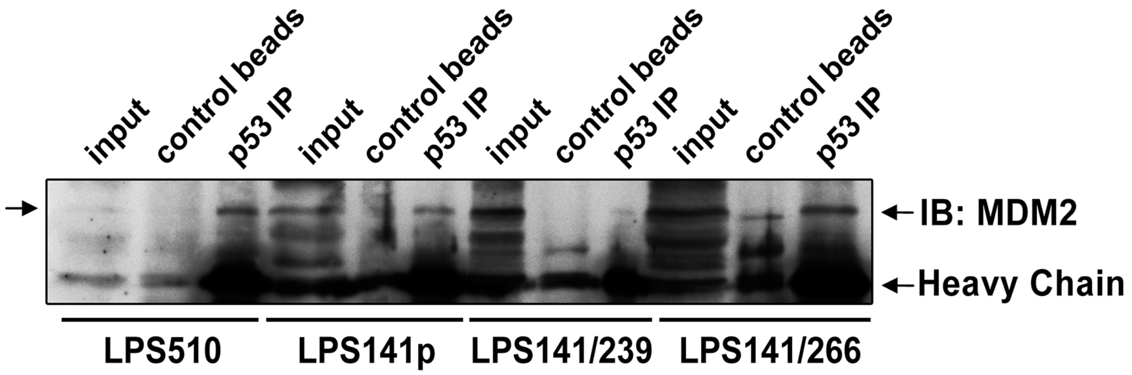

Supplementary Figure 1: MDM2-p53 interaction evaluated by p53 immunoprecipitation followed by MDM2 immunoblotting.

Supplementary Table 1: Cell apoptosis analyses (%), as shown in Figure 3F, after LBH and SAHA treatment

Inhibitor	DMSO				LBH (0.25 µM)				SAHA (5 µM)			
Cell lines	UL	UR	LL	LR	UL	UR	LL	LR	UL	UR	LL	LR
**LPS141**	5.8	8.6	83.3	2.4	4.6	17.1	75.1	3.2	10.3	17.1	71.3	1.3
**LPS141/239**	4.3	10.8	84.2	0.8	4.4	13.9	77.0	4.7	4.3	19.2	71.8	4.6
**LPS141/266**	4.2	10.2	80.9	4.7	3.3	9.8	81.0	5.9	3.7	15.5	72.3	8.5
**LPS510**	4.9	7.1	87.1	0.9	5.6	14.4	70.3	9.7	6.8	10.3	80.7	2.2
**LPS778**	9.3	7.4	77.6	5.7	8.1	22.7	54.6	14.6	5.7	23.0	55.3	16.1

Supplementary Table 2: Cell cycle analyses (%), as shown in Figure 3G, after LBH and SAHA treatment

Inhibitor	LPS141				LPS141/266				LPS141/239				LPS778				LPS510			
	$G_{1/0}$	G_2	S	A	$G_{1/0}$	G_2	S	A	$G_{1/0}$	G_2	S	A	$G_{1/0}$	G_2	S	A	$G_{1/0}$	G_2	S	A
**DMSO**	61	27	12	0	66	28	6	0	57	33	9	1	66	20	13	1	46	28	10	16
**LBH (0.25 µM)**	31	41	18	10	29	65	4	2	27	64	6	3	58	27	7	8	45	26	7	22
**SAHA (5 µM)**	41	43	15	1	66	29	4	1	53	41	5	1	78	14	6	2	46	31	5	18

A indicates apoptosis before G1 peak.

Supplementary Table 3: Cell cycle analyses (%), as shown in Figure 4D, after *MDM2* and *p53 shRNA* treatment

Cell lines	LPS141				LPS141/266				LPS141/239				LPS510			
**shRNAs**	$G_{1/0}$	G_2	S	A	$G_{1/0}$	G_2	S	A	$G_{1/0}$	G_2	S	A	$G_{1/0}$	G_2	S	A
**pLKO**	49	31	17	3	74	22	4	0	62	23	12	3	62	6	13	19
**MDM2 shRNA1**	73	17	7	3	70	23	6	1	41	31	17	11	56	4	9	31
**MDM2 shRNA2**	69	18	8	5	67	29	4	0	51	26	15	8	52	6	7	35
**p53 shRNA1**	50	30	18	2	68	25	6	1	45	35	15	5	63	12	11	18
**p53 shRNA2**	51	30	16	3	67	28	5	0	38	34	16	12	59	13	6	22
**MDM2 shRNA1 + p53 shRNA1**	54	28	17	1	65	29	5	1	40	31	12	17	55	12	5	28

A indicates apoptosis before G1 peak.
